# Between-Group Variation in Female Dispersal, Kin Composition of Groups, and Proximity Patterns in a Black-and-White Colobus Monkey (*Colobus vellerosus*)

**DOI:** 10.1371/journal.pone.0048740

**Published:** 2012-11-07

**Authors:** Eva C. Wikberg, Pascale Sicotte, Fernando A. Campos, Nelson Ting

**Affiliations:** 1 Department of Anthropology, University of Calgary, Calgary, Alberta, Canada; 2 Department of Anthropology and Institute of Ecology and Evolution, University of Oregon, Eugene, Oregon, United States of America; The University of Texas at San Antonio, United States of America

## Abstract

A growing body of evidence shows within-population variation in natal dispersal, but the effects of such variation on social relationships and the kin composition of groups remain poorly understood. We investigate the link between dispersal, the kin composition of groups, and proximity patterns in a population of black-and-white colobus (*Colobus vellerosus*) that shows variation in female dispersal. From 2006 to 2011, we collected behavioral data, demographic data, and fecal samples of 77 males and 92 females residing in eight groups at Boabeng-Fiema, Ghana. A combination of demographic data and a genetic network analysis showed that although philopatry was female-biased, only about half of the females resided in their natal groups. Only one group contained female-female dyads with higher average relatedness than randomly drawn animals of both sexes from the same group. Despite between-group variation in female dispersal and kin composition, female-female dyads in most of the study groups had higher proximity scores than randomly drawn dyads from the same group. We conclude that groups fall along a continuum from female dispersed, not kin-based, and not bonded to female philopatric, kin-based, and bonded. We found only partial support for the predicted link between dispersal, kin composition, and social relationships. In contrast to most mammals where the kin composition of groups is a good predictor of the quality of female-female relationships, this study provides further support for the notion that kinship is not necessary for the development and maintenance of social bonds in some gregarious species.

## Introduction

Sex bias in natal dispersal has major implications for the evolution and expression of female-female and male-male social relationships [1], [2], [3], [4]. The philopatric sex resides with same-sexed kin throughout its life and has the opportunity to gain both direct and indirect fitness benefits by forming long-lasting, affiliative relationships with familiar kin of the same sex [3], [5].

For example in many cercopithecines (e.g. baboons, macaques, and vervets), males show obligate dispersal and females remain philopatric or form new groups with matrilineal kin via group fissioning [6], [7]. Females form strong relationships with matrilineal female kin [8], [9], [10], and they rely on kin support for acquiring and maintaining their dominance rank [11], [12], [13], [14], [15], [16]. In contrast, matrilineal kinship has a relatively small impact on male-male social relationships in these species [6]. This pattern of female philopatry, female kin-based groups, and/or strong bonds among female kin was thought to be the typical pattern among gregarious mammals, partly because early evidence of sex-bias in dispersal was limited to long-term behavioral observations of a relatively small number of species [17], [18].

Instead of dispersal being a sex-specific characteristic of a species [1], recent research in birds and mammals paints a more complex picture wherein individuals of each sex may adjust their dispersal strategies according to local ecological and social conditions (e.g. *Acrocephalus sechellensis* [19], *Cervus elaphus* [20], *Ateles belzebuth* [21], *Propithecus edwardsi* [22], and *Gorilla beringei beringei* [23]). This leads to cases where the more philopatric sex may disperse at low rates, the more dispersed sex may occasionally remain philopatric, and some animals may disperse in parallel with kin (e.g. *G. b. beringei* [23], *Rhinopithecus roxellana* [24], *Cebus capucinus* [25], *Alouatta pigra* [26], *Chlorocebus pygerythrus* [27]). As a result, individuals belonging to the more philopatric sex may not always co-reside with same-sexed kin, while the opposite may be true for some individuals of the dispersing sex. Thus, the observed sex-bias in dispersal might not always lead to the expected pattern of kin composition of groups (e.g. *Tursiops aduncus* [28]). It is therefore important to document not only the variation around the typical dispersal pattern [4], but also how this variation affects the kin composition of groups and social relationships [7]. This is a virtually unexplored topic [7].

Our study species, *Colobus vellerosus* (ursine colobus or white-thighed black-and-white colobus), is an Old World monkey that is closely related to *Colobus guereza* (guerezas) and *Colobus polykomos* (western black-and-white colobus) [29], [30]. Because the frequency of female dispersal varies among species of black-and-white colobus, this genus provides an opportunity to investigate how variation in dispersal affects the kin composition of groups and social relationships while controlling for phylogenetic relationships. In this paper, we investigate the link between dispersal patterns, kin composition of groups, and a proxy of social relationships (proximity) in *C. vellerosus*. Our research team previously documented obligate male dispersal and facultative female dispersal in this species [31], [32]. However, the frequency of female dispersal and its impact on the kin composition of groups and social relationships remains unknown [33]. Here, we evaluate three possible scenarios that could fit our study population by combining demographic, behavioral, and genetic data. We base our predictions regarding dispersal on the patterns observed in *C. guereza* [34] and *C. polykomos* [35]. Hypothesis one: Males are obligate dispersers and females are predominantly philopatric [34], groups are female kin-based and female bonded [2], [36]. Hypothesis two: Males are obligate dispersers and females are predominantly dispersed [35], groups are not kin-based, and groups are male-female bonded [2], [36]. Hypothesis three: Due to between-group variation in female immigration in our study population, some groups will be female philopatric, female bonded and female kin-based (as predicted by hypothesis one) and other groups will be female dispersed, not kin-based, and male-female bonded (as predicted by hypothesis two). The predictions for hypotheses one and two are listed in Table 1. In the discussion, we will compare our findings with published data from the other two closely related species of colobus to further investigate how variation in dispersal affects social relationships in this genus. Because some terms used in this paper may have different meanings for different disciplines [7], we provide a set of definitions for the terms used in this paper (Table 2).

**Table 1:** Predictions for hypothesis one (female philopatry, female kin-based groups, and female bonded groups) and hypothesis two (female dispersal, not kin-based groups, and male-female bonded groups).

Dispersal	Kin composition		Social relationships
**Male-biased dispersal with obligate male dispersal and** **predominant female philopatry:**	**Female kin-based groups:**		**Female bonded:**
**a)** females reside longer than males in their natal group*;**b)** females reside longer than males in any given group towhich they belong*; **c)** more males than females will immigrateand emigrate during our study#; and **d)** more females than malesare classified as natal while more malesthan females are classified as immigrants#.	**a)** female-female but not male-maledyads have higher mean *R* within groupsthan between groups*; and **b)** female-femalebut not male-male dyads have highermean *R* than randomly drawn dyadsfrom the same group#.		**a)** female-female dyads (but not male-male or male-female dyads) have higher mean proximity scores than randomly drawn dyads from the same group#.
**No sex bias in dispersal with obligate male dispersal and** **predominant female dispersal:**	**Not kin-based groups:**	**Male-female bonded:**	
**a)** no tenure difference between males and females intheir natal group; **b)** no tenure difference between malesand females in any given group to which they belong; **c)** nodifference in number of males and females immigratingand emigrating during our study#; and**d)** no difference in number of males and femalesthat are classified as natal or immigrant#.	**a)** male-male and female-femaledyads do not have higher mean *R* withinthan between groups; and **b)** same-sexeddyads do not have higher mean *R* than randomlydrawn dyads from the same group#.	**a)** male-female dyads (but not male-male or female-female dyads) have higher mean proximity scores than randomly drawn dyads from the same group.	

* Supported in this study.

# Partly supported in this study.

**Table 2:** Definitions for terms used throughout this paper.

Term	Definition
**Breeding dispersal**	Animals emigrate from a social group where they have bred [1].
**Dispersal**	Animals emigrate from one social group and immigrate into another [1]. The term “biased” is used to describe the relative frequencies of male and female dispersal. For instance, male-biased dispersal means that more males than females disperse, but it does not indicate the degree of sex bias.
**Bonded groups**	Indicates strong social relationships within groups, either between males (male bonded), between males and females (male-female bonded), or between females (female bonded) [19].
**Immigrant**	An animal that resides in a different social group than where it was born.
**Kin-based groups**	Groups consist of related animals of the same sex. Female kin-based groups consist of mostly female kin and male non-kin. Male kin-based groups consist of mostly male kin and female non-kin [48].
**Natal**	An animal that resides in the social group where it was born.
**Natal dispersal**	Animals emigrate from the social group where they were born before they start to breed [1].
**Parallel dispersal**	Animals transfer between groups together, or they disperse singly to groups that already contain kin or other familiar animals (i.e. animals with whom they co-resided with at some point) [49].
**Philopatry**	Animals remain in the group where they were born [1]. Female-biased philopatry means that more females than males remain in their natal groups.

## Methods

### Study Site, Species, and Subjects

Boabeng-Fiema Monkey Sanctuary (BFMS) is located in central Ghana (7° 43′ N and 1° 42′W). BFMS contains 1.92 km^2^ of dry, semi-deciduous forest [37] that is connected to other forest fragments by narrow, riparian forest corridors. BFMS and surrounding forest fragments contain 28 groups of colobus, and some dispersal may occur between the fragments [38].

Sicotte and her students have studied the colobus at BFMS since 2000. At BFMS, the colobus reside in uni- or multi-male, multi-female groups with 9 to 38 animals [38]. Our research team previously concluded that males are obligate dispersers and females are facultative dispersers [39], [31]. These conclusions were partly based on group counts because not all females were individually recognized. Therefore, we could not determine the proportion of immigrant versus natal females, and we could not investigate between-group variation in dispersal and its impact on the kin composition of groups and social relationships.

To investigate these unexplored topics, we observed eight groups residing in the largest fragment (Boabeng). Observers recognized all animals in the study groups by the shape of their eye brows, body size, and sex. The numbers of adult males (>7 years), subadult males (3–7 years), adult females (>5 years), and subadult females (3–5 years) in each study group are presented in Table 3. We did not include infants and juveniles (0–3 years) because dispersal is rare before this age [32] while mortality rates are high [40]. Thus, animals that disappeared when younger than three years are likely to have died rather than dispersed. Hereafter, the term males and females will refer to adult and subadult animals unless otherwise specified.

**Table 3:** The study periods, contact hours, and the number of adult and subadult animals.

Group	Study period	Contact hours	Number of males	Number of females
**BO**	2008–2010	406	1–3	8–11
**BS**	2006–2011	406	1–8	6–7
**DA**	2006–2010	583	3–9	7–14
**NP**	2007–2011	371	1–4	5–6
**OD**	2006–2010	465	1–8	6–12
**RT**	2006–2011	613	3–4	6–8
**SP**	2006–2011	479	1–4	4–5
**WW**	2006–2011	644	4–6	7–17

### Behavioral Data Collection and Analyses

Demographic data were collected at least once a month from each study group when ECW was present at the site: 2006 (3 mo), 2007 (3 mo), 2008–2009 (12 mo), 2010 (1 mo), and 2011 (1 mo). We occasionally contacted four neighboring groups to determine the target group of study animals that had dispersed. When an animal’s dispersal status is known from the demographic records, we refer to it as “known immigrant” or “known natal” (see Appendix S1). We used demographic data collected between 2000 and 2005 from four study groups (BS, DA, RT, and WW groups) to determine the natal group of 10 study animals that were born during this time period [39], [31]. To analyze sex-bias in dispersal, we only used the dispersal events that occurred between 2006 and 2011 because all animals in the study groups were individually recognized during this time period.

We investigated observed sex bias in dispersal using two types of analyses. First, we used survival analyses to investigate: a) if the age at natal dispersal differed between males and females; and b) if the length of tenure differed between adult males and adult females. For the survival analyses, we used log-rank tests to evaluate statistically the difference between males and females. The log-rank test calculates the survival functions for males and females by summing the observed and expected frequencies of dispersal for each sex at each time interval. To perform the survival analyses, we used the package “Survival” [41] in R 2.13.2 [42]. Second, we used Fisher’s and chi-square tests with Yates’ correction to examine if males or females immigrated to or emigrated from their current group more often than expected by chance. We calculated the expected frequencies for males (or females) as the total number of events for males and females multiplied by the proportion of males (or females).

During 2008 and 2009, we collected 4993 group scans [43] from 110 animals with an average of 46 scans per animal (range: 13–94). We allocated 10 minutes each hour to locate visible animals to scan in the current focal group. During each scan, we recorded the identity of the scanned animal and all the other animals within one meter. We use a short distance category because groups are cohesive, and we believe that our estimates of this distance are precise because we can use the length of their tails (which is just under one meter long) as a reference [44], [45]. We could not identify all animals within 1 meter in 43 of the scan samples, and these samples were omitted from our analyses. We calculated the proximity score for animals A and B as the mean of the proportion of A’s scan samples in which B was within one meter and the proportion of B’s scan samples in which A was within one meter. We analyzed dyadic proximity scores with resampling procedures because this type of statistical test makes fewer assumptions regarding independence and distribution of the data than parametric statistical tests [46]. Similar resampling procedures have been used for comparisons across different age-sex classes in other studies [21], [47], [48]. We compared the observed average for female-female dyads in each study group with the average for same-sized simulated groups. The simulated groups consisted of dyads randomly drawn without replacement from all age-sex classes in the original group, and the simulated groups contained the same number of dyads as the number of female-female dyads in the original group. To evaluate if the observed average was significantly different from random, we used 10,000 iterations to generate the 95% and the 99% confidence intervals for the simulated averages. The resampling procedure was repeated for male-male and male-female dyads. The resampling procedures were conducted in Microsoft Excel 2010 using VBA code written by FAC (Appendix S2).

### Genetic Data Collection and Analyses

We collected two to five fecal samples on different occasions from each animal in the study groups. For each sample, we mixed approximately 1–2 g feces in 6 ml of RNAlater®. We stored the samples in a refrigerator (4°C) for up to twelve months before transferring them to a deep freezer (−20°C). For details regarding the laboratory protocols and primers, see Appendix S1. In brief, we extracted DNA from the fecal samples with the QIAamp DNA Stool Mini Kit. The amount of DNA in each extract was quantified using real-time PCR on a Roche Lightcycler 480 (Morin 2001). We amplified 20 short tandem repeat (STR) loci using human MapPair® primers. The PCRs were set up using Qiagen’s multiplex PCR kit with a modified protocol and run on an ABI Veriti® thermocycler. We electrophoresed the amplification products on an ABI 3730 DNA analyzer, and their sizes were evaluated against a size standard. Allele sizes were assigned by Genemapper v3.7, but also confirmed by visual inspection of the spectrograms. Based on the quality of the extracts, we determined the number of replicates needed to confirm allele sizes in homozygotes following Morin and colleagues’ method [49] but with locus-specific dropout rates for our study population. Heterozygotes were confirmed using two replicates. We used the multiple tubes approach for confirming all genotypes [50]. To control that the samples were correctly identified, we genotyped at least two samples per animal.

We computed dyadic estimates of relatedness (*R*) using the software COANCESTRY [51] following a method described in the Supporting Information (Appendix S1) and in Rollins and colleagues’ paper [52]. We used these *R* values to determine the “likely” dispersal status of 39 of 61 females whose status was unknown from the demographic record because they were already present in the groups when the study started. We used a genetic network method that assigns dispersal status to animals based on the distribution of close kin across groups [52]. We modified the genetic network method [52] to better fit the breeding system of our study species. We defined close kin based on the *R* for known kin, keeping in mind that we were interested in detecting events of female rather than male dispersal because we already knew the dispersal status of all males. We defined dyads with *R* above 0.43 as close kin because this is the lowest *R* for known parent-offspring and full-siblings (N = 55). Female-female dyads with *R* above this threshold were likely to be maternal kin, while we could not always tell if dyads with lower *R* were maternal kin, paternal kin, or non-kin. The range of *R* for close kin did not overlap with that of 45 non-kin (*R*: 0–0.20). We categorized an animal as a likely immigrant if it had a lower number of close kin in its current group than in any other group. We did not count co-residing offspring because the parent could have immigrated into the group before the offspring was born. In cases where an animal had the same number of close kin in its current group as in another group, we assigned it as: a) likely natal if the close kin in its current group were known or likely natal and the close kin in the other group were known or likely immigrant; and b) likely immigrant if the close kin in its current group were known or likely immigrant and the close kin in the other group were known or likely natal. We visualized this network of close kin using the package “igraph” [53] in R 2.13.2 [42]. Following the terminology used in network analysis, we will use the term genetic tie to refer to the link between two animals with *R* above 0.43. We evaluated the accuracy of the genetic network method in our dataset by comparing likely dispersal status (from the genetic network) and known dispersal status (from the demographic record) of 47 animals of all age-sex classes that were genotyped at more than 10 loci. We then combined information on known and likely dispersal status to test if there was a significant difference between observed and expected numbers of: a) natal males and females using Fisher’s exact test; and: b) immigrant males and females using chi-square tests with Yates’ correction. Expected numbers were calculated based on the proportion of males and females in our study. For example, we calculated the expected numbers of male immigrants as the total number of immigrant males and females multiplied by the proportion of males. All tests were two-tailed and significance was set to α = 0.05.

We investigated the kinship structure of groups using two kinds of simulations. First, we used simulations on a population level to see if average within-group *R* (i.e. average across the groups’ average *R*) for male-male dyads (or female-female dyads) differed from same-sized groups consisting of males (or females) randomly drawn from the population (VBA code available in Appendix S3). For the population analysis of female *R*, we included all females residing in the study groups in 2008. For the population analysis of males, we restricted our analysis to animals residing in three multi-male groups (BS, SP, and WW groups) in 2008. We had to exclude one of the four males in WW group due to low DNA extract quality and incomplete genotyping. Apart from this male, all the subadult and adult animals in these three groups were genotyped. We did not include the remaining multi-male groups in the analysis (DA, OD, and RT groups) because we did not genotype the majority of the resident males due to low DNA extract quality. Second, we compared the average *R* of male-male, male-female, and female-female dyads in each group with the average *R* of same-sized simulated groups of randomly drawn animals from the same group (VBA code in Appendix S2). For the group level analysis, we only included the three multi-male groups where the majority of males were genotyped. For both analyses, we used 10,000 iterations to evaluate if the observed groups were significantly different from the simulated groups.

This project compiled with the rules of the Animal Care Committee at the University of Calgary (Permit Number: BI 2006–28, 2009–25) and the laws of Ghana. The Ghana Wildlife Division and the management committee of the Boabeng-Fiema Monkey Sanctuary provided permission to conduct this study.

## Results

### Observed Dispersal

**Figure 1:**
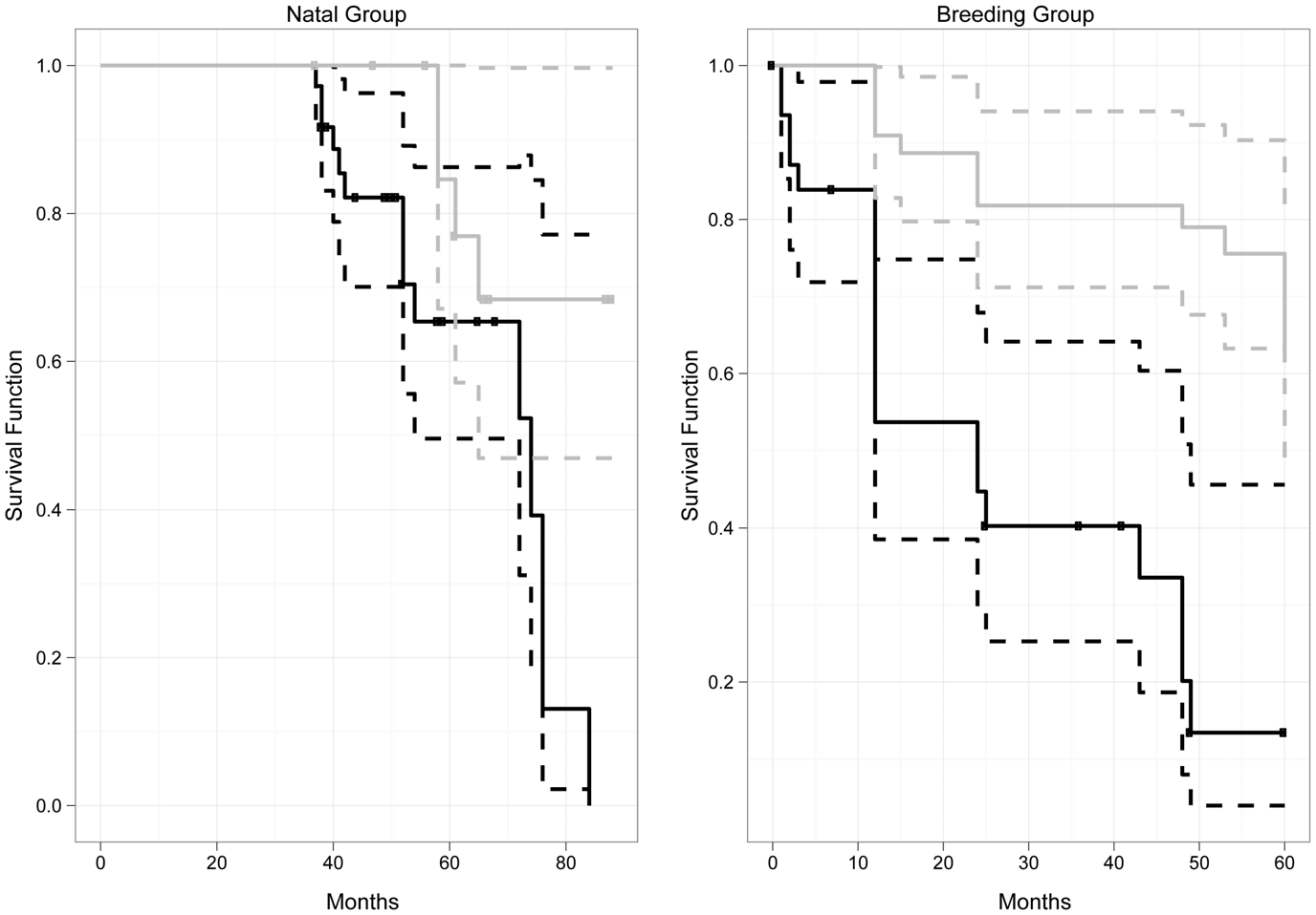
Male and female tenures in natal group and breeding group. The solid lines represent the survival function (i.e. probability of remaining in the current group) and the dotted lines represent the 95% confidence intervals. Dots indicate censored values. Males and females are represented by black versus gray lines.

Thirty-eight known natal males survived until they were at least three years old. Of these males, 23 still remained in their natal groups as subadults at the end of the study (age between 30 and 72 months). The other 15 natal males dispersed when they were between 37 and 84 months, and none of the males remained in their natal groups past adulthood. Twenty-one known natal females survived past the age of three years. Four of 21 females dispersed from their natal groups when they were between 58 and 65 months. The females that still resided in their natal groups at the end of the study were between 37 and 88 months old. According to the survival analysis, the median age for male natal dispersal was 74 months. Because less than half of the females dispersed before the end of the study, the survival analysis could not generate the median age for female natal dispersal. Females had a significantly longer tenure in their natal groups than males (Figure 1, log-rank test, chi-square = 5.9, df = 1, N_Males_ = 36, N_Females_ = 20, p<0.05).

The tenure of adult males ranged from 1 to over 60 months, while adult female tenure varied from 12 to over 60 months. The tenures over 60 months are censored, and we did not observe the start and/or the end of the tenure either because the animals were already residing in the group at the start of the study or because they remained in the group at the end of the study. The total number of censored values was 12 for males and 45 for females. The median tenure for adult males was 24 months. The median tenure for adult females could not be calculated because less than half of the females dispersed before the end of the study. Adult females had significantly longer tenure than adult males (Figure 1, log-rank test, chi-square = 25, df = 1, N_Males_ = 33, N_Females_ = 45, p<0.001).

**Figure 2:**
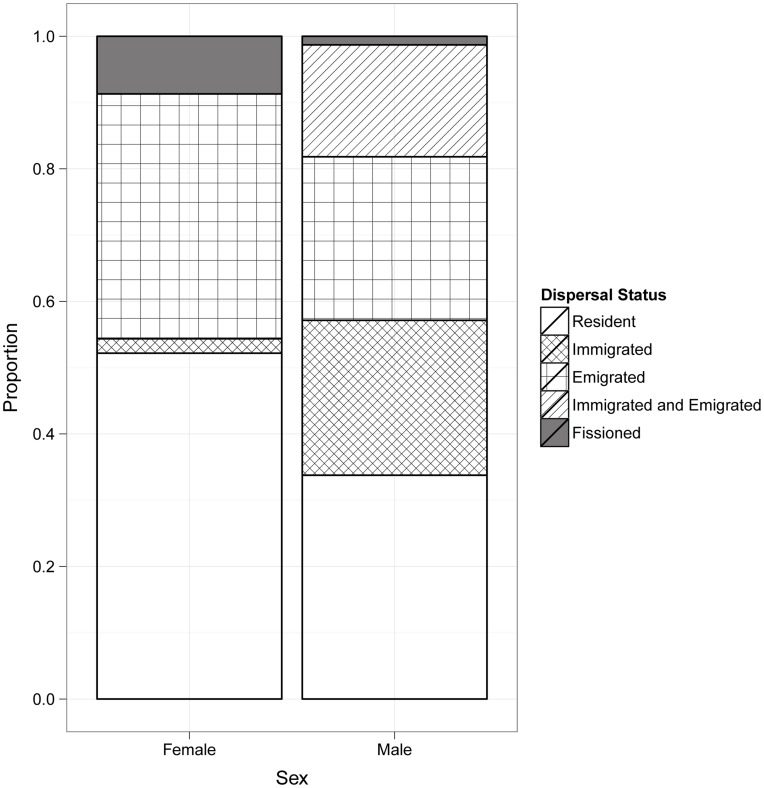
The proportion of males and females with different dispersal status.

Of the 77 males and the 92 females that resided in the study groups between 2006 and 2011, many remained in their initial group throughout the study (Figure 2). During this time period, a similar number of males immigrated (N = 40) and emigrated (N = 42). Two females immigrated into already existing study groups, whereas 34 females emigrated from the study groups. When comparing observed and expected frequencies of immigration and emigration, males immigrated more often than expected by chance (Fisher’s exact test, df = 1, p<0.01), while there was no difference in male and female emigration (chi-square = 1.4, df = 1, p = 0.23).

### Inferred Dispersal Status from Genetic Networks

**Figure 3:**
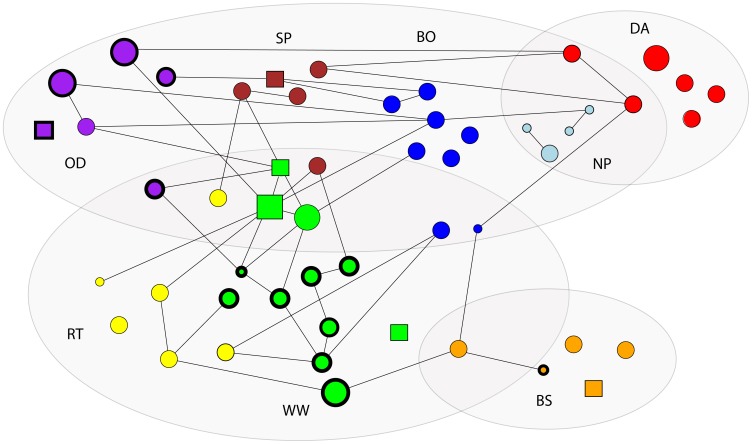
The genetic ties among adults. The shape specifies sex: circle = female and square = male. Thick borders indicate natal animals. Color indicates group. The size of the squares and circles refers to generation: large = first, midsized = second, and small = third generation. Shaded areas are genetic clusters of animals.

We organized the study animals into four clusters based on their genetic ties (i.e. *R* above 0.43) (Figure 3). For ease of viewing Figure 3, we only included animals that were adults at the start of the study. At least one animal from each cluster showed genetic ties to animals in other clusters, indicating likely dispersal events between clusters. Some clusters contained multiple groups, suggesting that some of these groups may have formed by a recent fission or parallel dispersal from the mother group. Based on genetic networks and observations, up to six cases of female parallel dispersal and/or group fission may have occurred in our study population, including 20 adult females in total. Not all of these females are depicted in Figure 3 because they were not adults when the study started. The remaining seven likely immigrant females in our study population may have dispersed singly. If subadult animals are included, all groups have more genetic ties within the group, and there are more genetic ties between DA and NP group.

We evaluated the accuracy of the genetic network method for assigning dispersal status in our dataset by comparing likely dispersal status (assigned from genetic networks) with known dispersal status (from demographic records) in 48 animals. The majority of the known natal animals and the known immigrant animals were correctly classified (Table 4). We could not determine the dispersal status for 33% of the known immigrants because they lacked close kin. One immigrant was incorrectly assigned as likely natal because she resided with her mother. In total, 83% of the animals with known dispersal status were correctly classified, 2% were incorrectly assigned, and 15% could not be assigned with the genetic network method.

**Table 4:** Assigned status via genetic networks for animals with known status and status based on genetic networks and/or demography for all animals.

	Assigned*		
Category	Likely natal	Likely immigrant	Unknown
**Known natal males and females**	100% (27)	0	0
**Known immigrant males and females**	5% (1)	62% (13)	33% (7)
	**Known or assigned** *		
**Category**	**Natal**	**Immigrant**	**Unknown**
**Adult females**	33% (16)	55% (27)	12% (6)
**Subadult females**	92% (11)	0	8% (1)
**Adult males**	0	100% (15)	0
**Subadult males**	93% (13)	7% (1)	0

#Counts presented in brackets.

* Assigned = assigned via genetic networks.

Next, we combined dispersal status information from demographic data (i.e. observed cases of dispersal and philopatry) and genetic networks. We were able to classify 86% of the females and 100% of the males confidently (Table 4). The remaining 14% of females did not have any close kin in the study groups, and we could not reliably determine their dispersal status. When comparing dispersal between adult males and adult females, a higher than expected number of females were classified as natal (Fisher’s exact test, df = 1, p = 0.01), while the observed numbers of male and female immigrants did not differ from expected (chi-square = 0.9, df = 1, p = 0.48). A similar number of subadult males and subadult females resided in their natal groups.

### Kin Composition

The percentage of female-female dyads that were close kin (*R*>0.43) varied among groups (NP: 20%, WW: 20%, BS: 19%, DA: 15%, SP: 13%, OD: 10%, RT: 7%, BO: 4%). All eight groups contained some female-female dyads with low *R* (0) and some with high *R* (>0.5), and female average within-group *R* ranged between 0.11 and 0.26. When we exclude subadult females, the average *R* was slightly lower in most groups. BO and SP groups did not contain any adult female close kin. We genotyped the majority of males in three groups (BS, SP, and WW groups). One adult male and two subadult males resided in BS group, and they were not close kin (*R*: 0.055–0.26). SP group consisted of one adult male that sired the two subadult males (*R*: 0.33–0.73), leading to a high percentage of male-male dyads being close kin (66%). WW group contained two adult males and one subadult male that were not close kin (*R*: 0–0.063).

**Figure 4:**
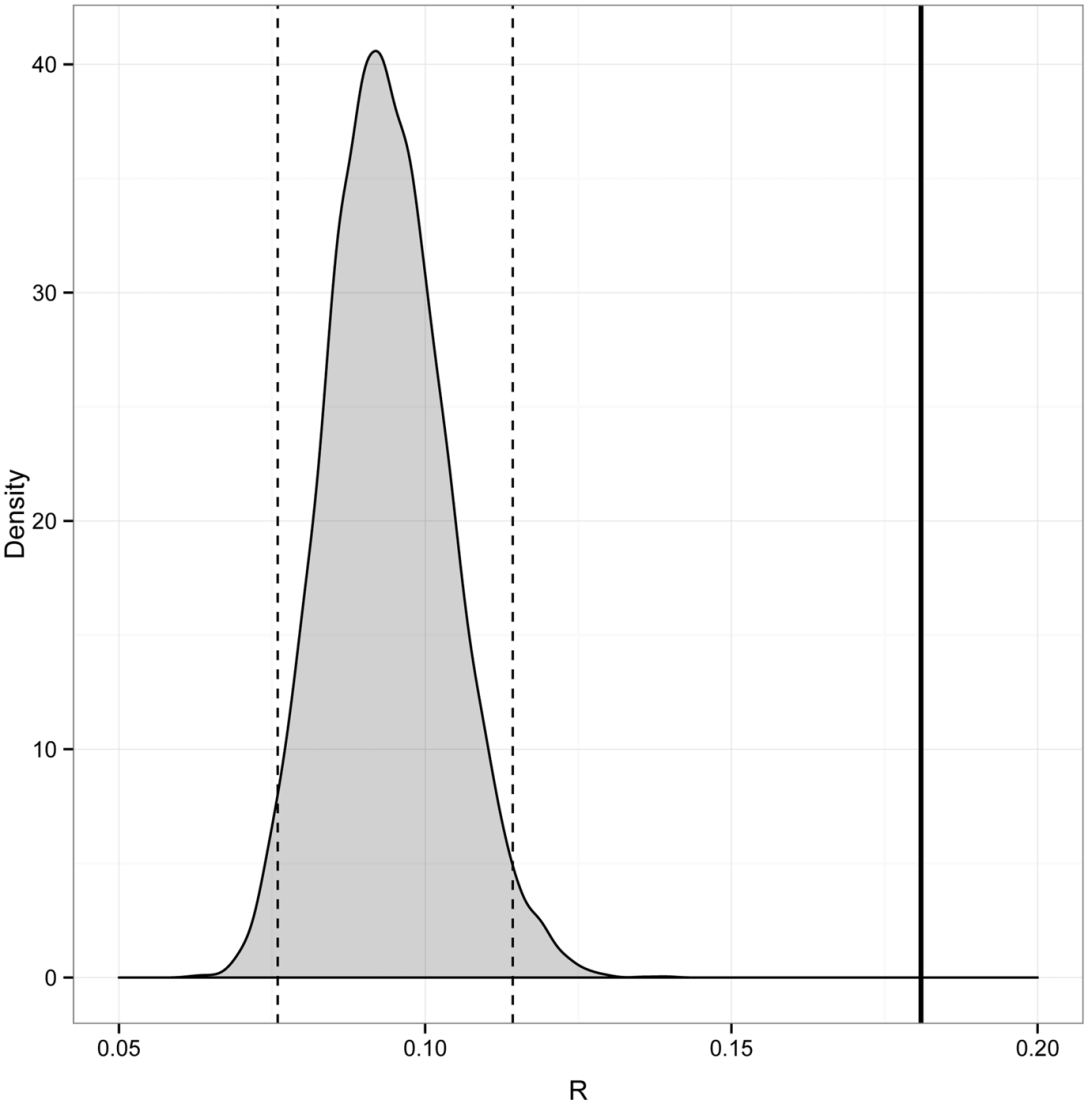
Female-female estimates of relatedness (*R*). Mean of mean female-female *R* (solid line) and simulated means (gray area) with 95% CI (dotted lines). The observed mean is calculated as the mean of the groups’ mean *R* values. The simulated groups consist of randomly drawn dyads from any of the groups.

On a population level, the observed average within-group *R* for females was significantly higher than the *R* for simulated groups consisting of females drawn at random from the population (Figure 4). We obtained similar results when excluding subadult animals: the observed average *R* for adult females (0.16) was significantly higher than the 99% confidence interval of simulated groups (0.07–0.14). The average within-group *R* for male-male dyads was 0.24, which is within the 95% confidence interval for simulated male-male dyads (0.041–0.32), and therefore not significantly different from random. We were not able to restrict our analysis to adult males since only one of the three groups contained multiple adult males.

**Figure 5:**
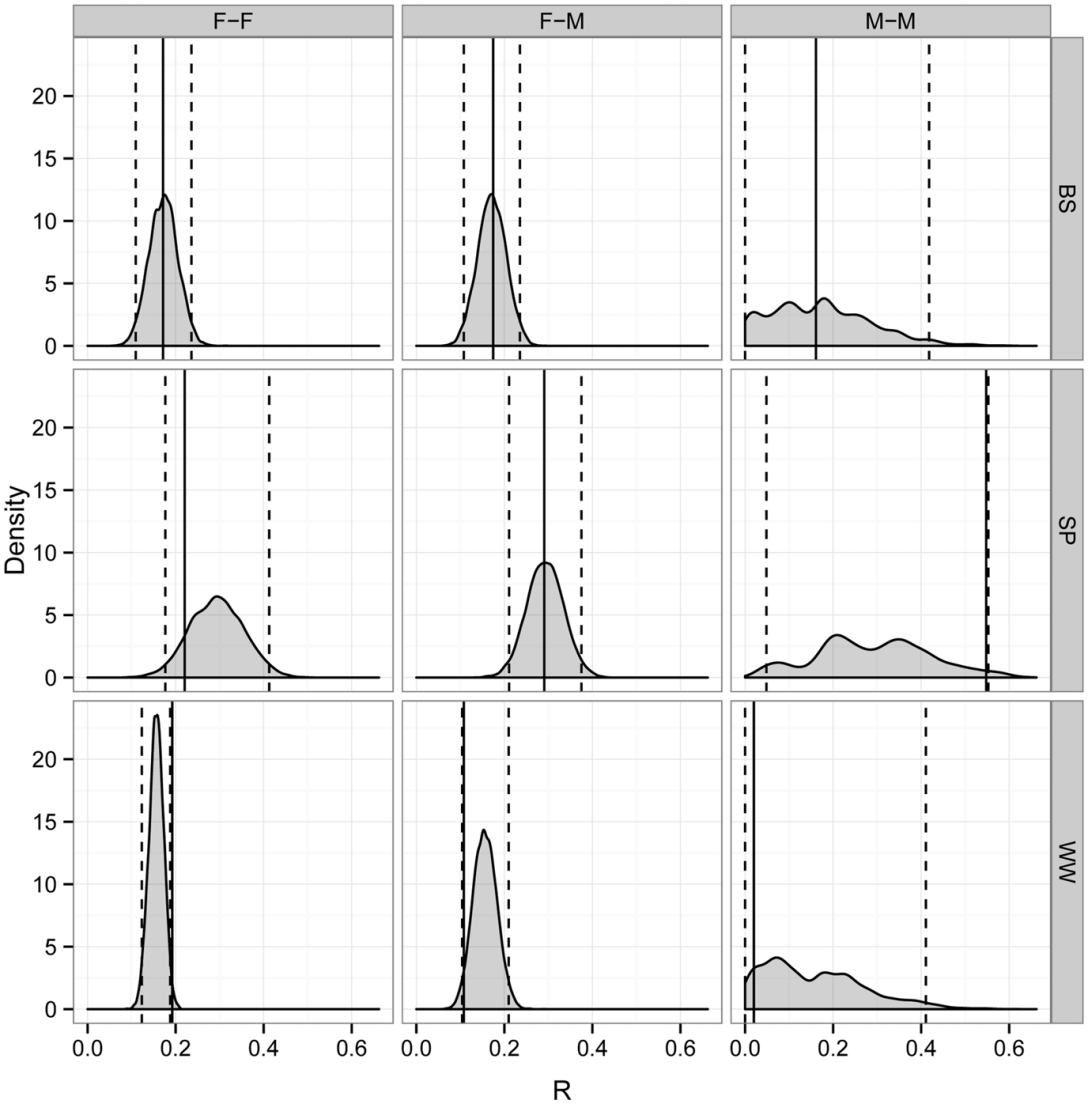
Within-group estimates of relatedness (*R*) for female-female, female-male, and male-male dyads. Observed mean within-group *R* (solid line) and simulated means (gray area) with 95% CI (dotted lines). Graphs are shown for female-female, female-male, and male-male dyads in three groups (BS, SP, and WW group). The simulated groups consist of randomly drawn dyads of any sex combination from the same group.

We performed similar simulations on a group level in the three groups where the majority of animals were genotyped (BS, SP, and WW groups). On a group level, female-female dyads in WW group showed higher average *R* than randomly drawn dyads from the same group at the 95% but not the 99% confidence level (Figure 5). No type of dyads (female-female, male-male, or male-female dyads) had higher average *R* than random in BS and SP group (Figure 5). In WW group, which contained more than one adult male, we also did the group level simulations using adults only. Adult female dyads in WW group had slightly higher average *R* (0.2176) than simulated groups at the 95% but not the 99% confidence level (95% CI: 0.106–0.2169).

### Proximity Patterns

Fifty-three of 61 females had higher average proximity scores with females than with males. Fifteen of 27 males residing in multi-male groups had higher average proximity scores with females than with males. In five of the seven multi-male groups (BS, DA, RT, SP, and WW group), female-female dyads had significantly higher average proximity scores than simulated groups, at least at the 95% confidence level (Table 5). In BO and OD group, no type of dyads had higher or lower proximity scores than simulated groups. Male-female dyads in three groups (BS, RT, and SP groups) had lower proximity scores than simulated groups (Table 5). When excluding subadult animals from the analysis, DA group showed higher average female-female proximity scores than simulated groups (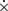: 0.043, 95% CI: 0.013–0.033). In the other three groups consisting of several adult males (BO, RT and WW groups), no type of dyad had higher proximity scores than simulated groups.

**Table 5:** Observed mean proximity scores and the 95% and 99% confidence interval for simulated groups.

Group		BO		BO		BO			BS		BS		BS		DA		DA		DA		NP	NP	NP
**Type of dyad** #		F-F		M-M		F-M			F-F		M-M		F-M		F-F		M-M		F-M		F-F	M-M	F-M
**Observed mean**		0.023		0.000		0.023			0.040*		0.024		0.019*		0.043**		0.005**		0.016*		0.041	–	0.032
**Simulated 99% lower CI**		0.018		0.000		0.011			0.018		0.000		0.018		0.013		0.011		0.016		–	–	–
**Simulated 95% lower CI**		0.019		0.000		0.013			0.021		0.004		0.021		0.014		0.013		0.017		–	–	–
**Simulated 95% upper CI**		0.027		0.096		0.033			0.037		0.063		0.038		0.029		0.029		0.025		–	–	–
**Simulated 99% upper CI**		0.028		0.104		0.036			0.040		0.075		0.040		0.031		0.032		0.026		–	–	–
**Group**	**OD**		**OD**		**OD**		**RT**			**RT**		**RT**		**SP**		**SP**		**SP**		**WW**		**WW**	**WW**
**Type of dyad**	F-F		M-M		F-M			F-F		M-M		F-M		F-F		M-M		F-M		F-F		M-M	F-M
**Observed mean**	0.031		0.029		0.021			0.026*		0.027		0.010**		0.077**		0.021		0.012**		0.020*		0.000	0.011
**Simulated 99% lower CI**	0.011		0.006		0.017			0.010		0.004		0.012		0.011		0.000		0.017		0.011		0.000	0.009
**Simulated 95% lower CI**	0.014		0.010		0.019			0.012		0.006		0.013		0.016		0.005		0.021		0.012		0.000	0.011
**Simulated 95% upper CI**	0.038		0.042		0.032			0.024		0.034		0.023		0.059		0.094		0.051		0.019		0.041	0.021
**Simulated 99% upper CI**	0.042		0.047		0.034			0.026		0.038		0.024		0.065		0.116		0.054		0.020		0.050	0.023

* Significant result at the 95% confidence level.

** Significant result at the 99% confidence level.

# F-F = female-female dyads, M-M = male-male dyads, and F-M = female-male dyads.

## Discussion

On a population level, the social structure of *C. vellerosus* conformed to some of the predictions listed under hypothesis one: male-biased dispersal, female kin-based groups, and female bonded groups (Table 1). However, our data were far from a perfect fit for this hypothesis due to substantial between-group variation in female dispersal, kin composition, and social bonds. We conclude that hypothesis three can best explain the patterns observed on a group level because groups fall on a continuum from female dispersed, not kin-based, and not bonded to female philopatric, kin-based, and bonded.

### Social Structure on a Population Level

Some of our results supported the predictions for male-biased dispersal while other results indicated a lack of sex-bias in dispersal. The contrasting results are likely linked to specific ecological and demographic characteristics of this population. The study groups reside in a relatively large forest fragment that has higher population density, larger groups, and higher habitat quality than surrounding, smaller fragments [38], [54], [55], [56]. Females that emigrate from the study groups may be unwilling or unable to immigrate into the large, neighboring groups [39]. Instead, they may have to disperse to smaller groups or establish new groups in the surrounding fragments with lower population densities. *Alouatta seniculus* (red howler monkeys) show a similar pattern, where females either gain a breeding position in their natal group or disperse [57]. The majority of dispersing howler females fail to join existing groups and sometimes have to disperse over long distances to find unoccupied habitat where they can establish a new group [57]. This particular pattern of female dispersal may explain why there was no sex bias in observed frequencies of emigration despite male-biased immigration.

The current conditions in our study population may also explain why we observed male-biased immigration during our study despite a lack of sex-bias in the number of likely immigrants residing in our study groups. The observed frequencies of male and female immigration reflect current conditions. In contrast, the genetic network analysis may reflect immigration events up to 20 years ago when groups were fewer and smaller in size [38], [55], [56], [58]. Since small group size increases the success of female immigration attempts, the genetic network analysis may reflect a past condition with more frequent female immigration.

Despite the variation in the intensity of sex bias depending on the variable of analysis, we conclude that the frequency of dispersal in this population is largely male-biased under current conditions because fewer males than females were classified as likely natal, and males changed groups more frequently than females. We would like to point out that this study as well as previous studies [39], [31], only investigated dispersal frequencies and not dispersal distances. Sometimes the patterns of sex bias in dispersal differ between these two variables [59], [60], [61]. Future studies should therefore investigate dispersal distance in addition to dispersal frequency, which may require relocating dispersing animals and sampling animals in the surrounding groups in the main fragment as well as surrounding fragments [61], [62], [63], [64].

Male-bias in dispersal was not coupled with predominant female philopatry as stated in hypothesis one. The numbers of immigrant and natal females were similar according to the genetic network analysis. Females in this population can therefore not be categorized as predominantly dispersed or predominantly philopatric. Because male-biased dispersal did not correspond with predominant female philopatry, we concur with Clutton-Brock and Lukas – it is important to report frequencies of dispersal *and* philopatry to understand the kin composition of groups [7]. Even if females were not predominantly philopatric, infrequent female immigration and regular male immigration made groups more likely to consist of female kin than male kin. Furthermore, most of the likely immigrant females dispersed in parallel or fissioned (Figure 3), and it is possible that females can continue living in a group with familiar close female kin despite dispersing. As expected under hypothesis one, female-female but not male-male dyads had higher average *R* within study groups compared to simulated groups of randomly drawn dyads from the population. Most females showed higher average proximity scores with females than males, which indicates that this population may be female-female bonded, also supporting hypothesis one.

### Social Structure on a Group Level

There was considerable between-group variation in social structure, which supports hypothesis three. Females in some groups were predominantly philopatric or predominantly dispersed, while some groups contained a similar number of likely natal and immigrant females. As expected for a species with variation in female dispersal, average within-group *R* varied between groups. Variation in average *R* is often explained by group size. A decreased proportion of kin to non-kin in larger groups (i.e. dilution of kin) often creates a negative correlation between average *R* and group size [65]. However, the dilution of kin effect cannot fully explain the observed between-group variation in our population because the two smallest groups showed the lowest and the highest average female *R*. Therefore, we suggest that additional factors related to group formation may affect average *R* among females, similar to *A. seniculus* [66] and *Marmota flaviventris* (yellow-bellied marmots) [67]. In these species, female immigration to established groups is rare, and groups increase in size largely due to the selective recruitment of female kin as breeding females. Therefore, large groups have higher average *R* than smaller groups [66]. In contrast to *A. seniculus* where newly established groups often consist of female non-kin [66], the average *R* in new groups varied in our population. SP group (formed in 2004) showed low average female *R*, while NP (formed in 2007 as a fission product of DA group) had high average female *R*. Based on these findings, we suggest that between-group variation in average *R* likely depends on a range of factors such as group size, age of groups, tenure, individuals’ reproductive histories in the group, and parallel dispersal.

We classified WW group as female kin-based because this was the only group that showed higher average *R* between females than randomly drawn dyads from the same group. This finding may be misleading as female average *R* in WW group was intermediate relative to BS and SP groups. We suspect that the groups were classified differently due to differences in kinship between the resident adult and subadult males. These subadult males will likely disperse before reaching adulthood. Unfortunately, we could not perform the simulations in BS and SP groups after excluding the subadult animals because both of these groups contained only one adult male.

In most groups, female-female dyads showed higher proximity scores than randomly drawn dyads from the same group when including subadult animals. Excluding the subadults, only females in DA group showed higher proximity scores than random. This finding indicates that in most groups, subadult females but not subadult males are often in proximity with adult females. This difference in social integration between natal males and females may reflect their dispersal patterns, as in *Mus musculus domesticus* (house mice) [68].

Neither dispersal patterns nor kinship were sufficient to explain the occurrence of strong female bonds. In contrast to our predictions, immigrant female non-kin in SP group showed the highest female-female proximity scores. It is possible that females maintain strong relationships with familiar females despite dispersing because a high proportion of the likely immigrant females resided with at least one female that originated from the same group (Figure 3). We also suspect that the formation of strong female social bonds is facilitated not only by kinship but by familiarity through long co-residency. This pattern is observed in *Equus ferus caballus* (feral horses), in which females disperse from their natal group and may remain for the rest of their lives in the new group that they enter [69]. In some species, it may be that individual selection rather than kin selection shapes cooperative and possibly reciprocal affiliative behaviors [5], [70]. These behaviors are particularly likely to occur when they lead to immediate rather than delayed benefits, which may be the case for communal resource defense and grooming [5], [70]. Future studies should further investigate the benefits that females may gain from forming strong relationships using more direct social behaviors such as grooming and infant handling. Such studies could detect if females maintain close proximity to each other because of bonds with other females or because they cluster around the same male [71].

Finally, we want to point out that the two groups with highest proximity scores had the smallest group size. Group size may affect proximity patterns inversely, as it may become harder for a female to keep close proximity with all other females as the number of partners and group spread increase. Each female may only be in close proximity with a small proportion of the females in large groups, which will lower the group’s mean proximity score. A large group size may explain why WW group that consists of several natal female kin had low mean proximity scores. However, group size is not the only factor influencing mean female-female proximity scores because the third largest group had the highest score, and this was the only group that was female-female bonded when excluding subadult animals from the resampling procedure.

### Are Groups Female Kin-based and Bonded?

Researchers often categorize populations according to the presence or absence of kin-based and bonded groups [2], [36]. However, populations with individual variation in dispersal are likely to exhibit a continuum from not female kin-based to highly female kin-based (or not bonded to highly bonded). Studies of red howlers [66] and yellow-bellied marmots [67] indicate that variation in female kinship may occur even within groups throughout time. In light of these issues, we summarized our results in two ways (Table 6). We used the results from the within-group simulations of *R* and proximity to determine which of the two categories provides the best fit for each group. We also compare mean *R* and proximity across groups to estimate how strongly kin-based and bonded groups were in relation to each other. None of the groups conformed to all predictions listed in hypothesis two. Although some groups were female dispersed and not kin-based, none of the groups were male-female bonded as *G. b. beringei* [71] and *Lagothrix poeppigii* (lowland woolly monkeys) are [47]. NP and WW groups may conform fully to hypothesis one (female philopatric, kin-based, and female bonded), and thus have a similar social structure as *Nasua narica* (white-nosed coatis) [72]. WW group conformed to all the predictions when including subadults in the analysis, but the group is not female bonded when excluding the subadults. NP group conformed well to hypothesis one with the exception that all females were classified as dispersers. All females in NP group fissioned from DA group. Because none of these females underwent social dispersal [73], they may be better described as philopatric. Likewise, BO, DA, RT, and SP groups may be classified as facultative female dispersed rather than truly dispersed because they consist of at least some females that did not undergo social dispersal (Figure 3). If classifying females that showed parallel dispersal or group fission as philopatric, these groups provided at least partial support for the predicted link between dispersal, kin composition, and social relationships. In support of hypothesis three, we conclude that groups fall on different ends of a continuum from female dispersed, not kin-based, and not bonded to female philopatric, kin-based, and bonded.

**Table 6:** Dispersal pattern, kin composition, and social relationships among adult females.

Group	Dispersal pattern	Kin-based#	Average *R*	Bonded#	Average proximity
**BO**	Dispersed	–	0.12 (intermediate)	No	0.019 (low)
**BS**	Facultative	–	0.16 (intermediate)	–	0.042 (high)
**DA**	Facultative	–	0.14 (intermediate)	Yes	0.043 (high)
**NP**	Dispersed	–	0.26 (high)	–	0.041 (high)
**OD**	Philopatric	–	0.18 (intermediate)	–	0.031 (intermediate)
**RT**	Dispersed	–	0.15 (intermediate)	No	0.026 (intermediate)
**SP**	Dispersed	–	0.07 (low)	–	0.100 (high)
**WW**	Philopatric	Yes	0.22 (high)	No	0.014 (low)

# Based on results from the within-group simulations.

Our classification of bondedness is based on a within-population comparison of social relationships. In contrast to other primate taxa such as macaques, female colobus show low rates of grooming [33] and may therefore be characterized as more weakly female bonded. Female *Macaca thibetana* (Tibetan macaques) show higher grooming rates despite having access to a similar number of female kin [74]. In this case, differences in the kin composition of groups cannot explain the between-species variation in social relationships, and other factors such as time constraints on social behaviors may be important in shaping social relationships.

### Comparison with Other Black-and-White Colobus

Because the frequency of female dispersal varies among species of black-and-white colobus, this genus provides an interesting opportunity to investigate if the species-specific dispersal pattern is sufficient to predict social relationships. Female *C. guereza* are predominantly philopatric [34], *C. polykomos* are predominantly female-dispersed [35], and we have shown that *C. vellerosus* show facultative female dispersal. Despite this variation in female dispersal, all three species show relatively strong female-female relationships compared to male-male and male-female dyads in the same group [33], [34], [35], [75]. In contrast to the expectations, *C. polykomos* with the highest frequency of female dispersal show the highest rates of grooming [35]. Based on these comparisons, the relative strength of female social relationships appears unaffected by the between-species variation in female dispersal. This comparison provides further support for the notion that kinship is not necessary for the development of social bonds, or alternatively, that dispersing females can continue residing with familiar female kin via parallel dispersal. This study demonstrates that dispersal patterns, the kin composition of groups, and social relationships are weakly linked in black-and-white colobus, and we encourage future studies to investigate these three variables simultaneously to enhance our understanding of the evolution of social structure.

## Supporting Information

Appendix S1 **Methods.** Methods for 1) determining dispersal status based on demographic data, 2) laboratory protocols, and 3) determination of allele sizes, computation of R-values, and kinship classification.
(DOCX)

Appendix S2 **VBA code for within-group simulations.** VBA code and sample data in Microsoft Excel for within-group simulations.
(XLSM)

Appendix S3 **VBA code for within-population simulations.** VBA code and sample data in Microsoft Excel for within-population simulations.
(XLSM)
