# Supplementary material for: Between-Group Variation in Female Dispersal, Kin Composition of Groups, and Proximity Patterns in a Black-and-White Colobus Monkey (Colobus vellerosus)
Source: PLoS One. 2012 Nov 7;7(11):e48740. doi: 10.1371/journal.pone.0048740 (PMC3492432; doi:10.1371/journal.pone.0048740)
Supplement: Appendix S1 — Methods. Methods for 1) determining dispersal status based on demographic data, 2) laboratory protocols, and 3) determination of allele sizes, computation of R-values, and kinship classification. (DOCX) [file pone.0048740.s001.docx]

**Appendix S1**

*Known dispersal status from the demographic database*

An animal’s dispersal status remained unknown if it was older than one year and already resident in a study group when first recognized. We considered animals as natal if they were less than one year old when we first recognized them. We classified animals that appeared in a study group after the start of the study as immigrants. We classified apparently healthy subadult and adult animals that disappeared from the study groups as emigrants. The predation risk is low for subadult and adult animals since large predators have been extirpated from the site and hunting is banned [[1](#_ENREF_1)]. During eleven years, we observed three males and two females die. The males died from wounds caused by intraspecific aggression, one female might have died of old age, and one female might have died of illness. Since the mortality risk for subadult and adult animals appears to be low, disappearances of young, healthy animals are likely due to dispersal rather than death.

*Protocols for DNA extraction, quantification, amplification, and size determination*

We extracted DNA from the fecal samples with the QIAamp DNA Stool Mini Kit following the manufacturer’s protocol with following modifications: 1) we lysed DNA samples in ASL buffer overnight after which they were centrifuged for three minutes; 2) we centrifuged samples for five minutes to pellet fecal matter and inhibitors; 3) we used 35μl Proteinase K to digest samples; 4) we incubated samples in AL buffer and Proteinase K for 30 minutes; and 5) we eluted DNA in 75μl AE buffer after a 30 minute incubation period.

We quantified the amount of DNA in each extract using a real-time polymerase chain reaction (PCR) protocol with a TaqMan® probe [[2](#_ENREF_2)], [[3](#_ENREF_3)]. The 20μl reactions were set up with 6.36μl H_2_O, 0.64μl MgCl_2_, 1μl oligo, 10μl LightCycler® 480 Probes Mastermix (Roche Applied Science catalog number 04707494001), and 2μl DNA template. The reactions were performed on a Roche Lightcycler 480 with the following cycling parameters: 1) initial incubation at 95°C for 10 minutes with a ramp temperature of 4.4°C, and 2) 50 cycles at 95°C for 10 seconds and 59°C for 20 seconds, with ramp temperatures of 4.4°C and 2.2°C respectively. Each set of reactions contained two replicates each of three positive controls with human DNA of known concentration and two negative controls with RNase-free water instead of DNA template. These controls were matched up with an external standard curve created from three replicates of five samples with known concentration and three negative controls. The correlation coefficient of the standard curve was 0.97. We calculated the average concentration for each extract from two to three replicates.

We amplified the following 20 short tandem repeat (STR) loci using human MapPair® primers: c19a, d1s207, d1s548, d1s1665, d3s1229, d3s1766, d4s243, d4s2408, d5s1457, d6s311, d6s474, d6s1056, d7s503, d10s611, d10676, d10s1432, d11s2002, d13s321, d14s306, and fesp [[4](#_ENREF_4)], [[5](#_ENREF_5)], [[6](#_ENREF_6)], [[7](#_ENREF_7)], [[8](#_ENREF_8)], [[9](#_ENREF_9)], [[10](#_ENREF_10)]. To amplify the STRs, we set up PCR reactions with 1 μl primer mix with each primer at a concentration of 10μM (using one or two primer pairs per reaction), 2.5μl multiplex master mix (Qiagen catalog number 206143), and 1.5μl of DNA. We included one negative control with RNase-free water instead of DNA template with each PCR. We amplified the STRs on an ABI Veriti® thermocycler using the cycling parameters listed in Qiagen’s Multiplex PCR Kit manual with the following modifications: 1) 55⁰C annealing temperature; 2) 60 second extension period; and 3) 35 cycles.

FAM or HEX fluorescently labeled 5’ ends of the forward primers made it possible to determine the size of the amplification products via capillary electrophoresis. We electrophoresed the amplification products on an ABI PRISM3730, and their sizes were evaluated against the GeneScan™ 500 ROX™ size standard (ABI catalog number 401734). Allele sizes were assigned by Genemapper v3.7, but also confirmed by visual inspection of the spectrograms.

We used the software Arlequin 3.1 to test for Hardy-Weinberg equilibrium and linkage equilibrium [[11](#_ENREF_11)], and we excluded 3 of the 20 loci from the analyses because of too many missing genotypes (d13s321), deviation from Hardy-Weinberg equilibrium (d10s611), or deviation from linkage equilibrium (fesp).

*Determining kinship*

We followed Rollins and colleagues’ [[12](#_ENREF_12)] method for defining known kinship using both observed pedigrees and parentage assignments in CERVUS [[13](#_ENREF_13)], [[14](#_ENREF_14)]. CERVUS calculates likelihood ratios and evaluates if a parent can be assigned with statistical confidence when taking into account the allele frequencies in the population, the proportion of candidate parents sampled, proportion of loci genotyped, and genotyping errors [[13](#_ENREF_13)], [[14](#_ENREF_14)]. The presence of full siblings in the candidate parent pool will reduce the power of the analysis. To mitigate this problem, we only included animals that are at least five years older than the offspring in the pool of candidate parents since this is the average age at which females become mature. Animals with an age difference greater than five years are unlikely to be sired by the same male since five years is longest observed male-tenure (this study). When group residency was known for the offspring, only parents who resided in the same group at the time of parturition were considered as candidate mothers. Since the proportion of sampled candidate parents varied depending on the offsprings’ ages, we divided our study animals into four age classes. The first age class included 25 young offspring that were less than five years old at the start of the study. For the young offspring, we sampled 90% of the candidate mothers and 10% of the candidate fathers. The second age class included eight mid-aged offspring that were between five and eight years at the start of the study. For the mid-aged offspring, we sampled 14 candidate mothers and none of the candidate fathers. We estimated that the number of sampled candidate mothers corresponds to approximately 79% of the females residing in the study groups at the time of their birth based on a “compete count” of the population from 2000 [[1](#_ENREF_1)]. The third age class included 19 older offspring (i.e. between 8 and 12 years at the start of the study), and we only sampled 5 candidate mothers which is approximately 31% of the adult females in the study groups at the time of their birth. The proportion of loci mistyped was set to 0.01. Only parents that were assigned with 95% confidence were considered true parents.

*Computing estimates of relatedness (R)*

We computed dyadic estimates of relatedness (*R*) using the software COANCESTRY [[15](#_ENREF_15)], which calculates *R* using two likelihood methods [[16](#_ENREF_16)], [[17](#_ENREF_17)] and five moment estimators [[18](#_ENREF_18)], [[19](#_ENREF_19)], [[20](#_ENREF_20)], [[21](#_ENREF_21)], [[22](#_ENREF_22)], [[23](#_ENREF_23)]. We investigated which of these methods was most accurate in our data set by correlating estimated (*R*) and actual relatedness (*r*) in 150 dyads with known kinship [[12](#_ENREF_12)] using Spearman rank correlations in R 2.13.2 [[24](#_ENREF_24)]. Because we obtained the highest correlation coefficient when using *R* values generated by Milligan’s [[16](#_ENREF_16)] dyadic likelihood estimator (Spearman’s r=0.90, df=148, p<0.001), we chose to use these *R* values for the analyses below.

**References**

1. Saj TL, Teichroeb JA, Sicotte P (2005) The population status of the ursine colobus (*Colobus vellerosus*) Boabeng-Fiema, Ghana. In: Patterson JD, Wallis J, editors. Commensalism and conflict: The human-primate interface. Norman, OK: The American Society of Primatology. pp. 351-377.

2. Heid C, Stevens J, Livak K, Williams P (1996) Real time quantitative PCR. Genomic Research 6: 986-994.

3. Morin P, Chambers K, Boesch C, Vigilant L (2001) Quantitative polymerase chain reaction analysis of DNA from noninvasive samples for accurate microsatellite genotyping of wild chimpanzees (*Pan troglodytes verus*). Mol Ecol 10: 1835-1844.

4. Arandjelovic M, Guschanski K, Schubert G, Harris TR, Thalmann O, et al. (2009) Two-step multiplex polymerase chain reaction improves the speed and accuracy of genotyping using DNA from noninvasive and museum samples. Mol Ecol Res 9: 28-36.

5. Morin PA, Mahboubi P, Wedel S, Rogers J (1998) Rapid screening and comparison of human microsatellite markers in baboons: Allele size is conserved, but allele number is not. Genomics 53: 12-20.

6. Bradley BJ, Boesch C, Vigilant L (2000) Identification and redesign of human microsatellite markers for genotyping wild chimpanzee (*Pan troglodytes verus*) and gorilla (*Gorilla gorilla gorilla*) DNA from faeces. Conserv Genet 1: 289-292.

7. St George D, Witte SM, Turner TR, Weiss ML, Phillips-Conroy J, et al. (1998) Microsatellite variation in two populations of free-ranging yellow baboons (*Papio hamadryas cynocephalus*). Int J Primatol 19: 273-285.

8. Coote T, Bruford MW (1996) Human microsatellites applicable for analysis of genetic variation in apes and old world monkeys. J Hered 87: 406-410.

9. Yamane A, Shotake T, Mori A, Boug AI, Iwamoto T (2003) Extra-unit paternity of hamadryas baboons (*Papio hamadryas*) in Saudi Arabia. Ethol Ecol Evol 15: 379-387.

10. Xiao H, Merril CR, Polymeropoulos MH (1992) Dinucleotide repeat polymorphism at the D3S1229 locus. Hum Mol Genet 1: 290.

11. Excoffier L, Laval G, Schneider S (2005) Arlequin ver. 3.0: An integrated software package for population genetics data analysis. Evol Bioinform 1: 47-50.

12. Rollins LA, Browning LE, Holleley CE, Savage JL, Russell AF, et al. (2012) Building genetic networks using relatedness information: a novel approach for the estimation of dispersal and characterization of group structure in social animals. Mol Ecol 21: 1727–1740.

13. Kalinowski ST, Taper ML, Marshall TC (2007) Revising how the computer program CERVUS accommodates genotyping error increases success in paternity assignment. Mol Ecol 16: 1099-1106.

14. Marshall TC, Slate J, Kruuk LEB, Pemberton JM (1998) Statistical confidence for likelihood-based paternity inference in natural populations. Mol Ecol 7: 639-655.

15. Wang J (2011) COANCESTRY: a program for simulating, estimating and analysing relatedness and inbreeding coefficients. Mol Ecol Res 11: 141-145.

16. Milligan BG (2003) Maximum-likelihood estimation of relatedness. Genetics 163: 1153-1167.

17. Wang J (2007) Triadic IBD coefficients and applications to estimating pairwise relatedness. Genet Res 89: 135-153.

18. Li CC, Weeks DE, Chakravarti A (1993) Similarity of DNA fingerprints due to chance and relatedness. Hum Hered 43: 45-52.

19. Lynch M (1988) Estimation of relatedness by DNA fingerprinting. Mol Biol Evol 5: 584-599.

20. Lynch M (1999) Estimating genetic correlations in natural populations. Genet Res 74: 255-264.

21. Queller DC, Goodnight KF (1989) Estimating Relatedness Using Genetic-Markers. Evolution 43: 258-275.

22. Ritland K (1996) Estimators for pairwise relatedness and individual inbreeding coefficients. Genet Res 67: 175-185.

23. Wang JL (2002) An estimator for pairwise relatedness using molecular markers. Genetics 160: 1203-1215.

24. R Development Core Team (2011) R: A language and environment for statistical computing. R Foundation for Statistical Computing. Available: <http://www.R-project.org>. Accessed 2010 Sep 21.
